# Comparative Analysis of the Methanogen Diversity in Horse and Pony by Using *mcrA* Gene and Archaeal 16S rRNA Gene Clone Libraries

**DOI:** 10.1155/2014/483574

**Published:** 2014-01-30

**Authors:** Khin-Ohnmar Lwin, Hiroki Matsui

**Affiliations:** ^1^Graduate School of Bioresources, Mie University, 1577 Kurimamachiya-Cho, Tsu, Mie 514-8507, Japan; ^2^Livestock Breeding and Veterinary Department, Yangon Diagnostic Laboratory, Yangon 11121, Myanmar

## Abstract

Comparative analysis of methanogen compositions in the feces of horse and pony was carried out by constructing the **α**-subunit of methyl coenzyme-M reductase (*mcrA*) gene and 16S ribosomal RNA gene (16S rRNA) clone libraries. The *mcrA* clone library analysis indicated that Methanomicrobiales was predominant in both horse and pony. Furthermore, most of the clones of the 16S rRNA gene library showed that Methanomicrobiales was also predominant in horse and pony, but the LIBSHUFF analysis showed that the horse and pony libraries were significantly different (*P* < 0.05). Most of operational taxonomic units (OTUs) showed low similarity to the identified methanogens in both the *mcrA* and the 16S rRNA clone libraries. The results suggest that horse and pony harbor unidentified and novel methanogens in their hindgut. The methanogen population was higher in horse than in pony; however, the anaerobic fungal population was similar in horse and pony. The methanogen diversity was different between two breeds of *Equus caballus*.

## 1. Introduction 

Members of the Equidae family, such as horse and pony, possess an anatomically specialized hindgut that accommodates a microbial ecosystem consisting of bacteria, protozoa, and anaerobic rumen fungi that are capable of degrading and fermenting structural polysaccharides of the plant cell walls [1]. Furthermore, methanogens that reduce CO_2_ with H_2_ to form methane are also common inhabitants of the hindgut of Equidae [2].

To date, many studies have been published on the microbial diversity in the hindgut of horse and pony. These studies include the diversity of bacteria [2–9], protozoa [3, 9], and anaerobic rumen fungi [3]. However, only limited information is available on the methanogen population in the hindgut of horses [10]. Many studies have been conducted to analyze the composition and population size of methanogens from the rumen of ruminants and other types of herbivores [11–24]. These studies have shown that methanogens that are affiliated to Methanobacteriales, Methanomicrobiales, and Methanoplasmatales  [25] are major constituents of the rumen of ruminants. Information about the methanogen density and diversity in the hindgut of horse and pony are important for fully understanding the microbial ecosystem in their hindgut.

The diversity in the microbial communities of the rumen is influenced by many factors, such as location, environment, feed composition, feeding frequency, supplements, animal species, and genetic background of individual animals within a species [26]. The composition of the microbiota in the hindgut of equine could depend on the hindgut capacity as a reflection of body size or even horse breed [9].

This study aimed to comparatively analyze the archaeal 16S rRNA gene and the *mcrA* gene in thoroughbred horses and Japanese local ponies kept under the same management.

## 2. Materials and Methods

### 2.1. Animals, Diets, and Sample Collection

Six mature thoroughbred horses and 3 mature Japanese local ponies were used for this study. Sex and age of each animal are as follows: horse 1 (gelding, 17), horse 2 (female, 12), horse 3 (gelding, 17), horse 4 (gelding, 12), horse 5 (gelding, 14), horse 6 (female, 14), pony 1 (gelding, 4), pony 2 (gelding, 10), and pony 3 (gelding, 7). Animals were fed regularly twice per day with Timothy hay, barley, and rice bran at a ratio of 2.0 : 1.5 : 1.0 kg for horses and 1.5 : 1.0 : 0.5 kg for ponies. Salt and calcium were used as supplements and water was available *ad libitum*. The animals are used for equestrian art and perform exercises every morning under the same management. To determine the microbial population and methanogen diversity, freshly voided fecal samples were collected and kept at 4°C during delivery to the laboratory and then stored at −25°C.

### 2.2. pH and Real-Time PCR Analysis of Total Bacteria, Methanogen, and Anaerobic Rumen Fungi Populations

Five grams of feces was mixed with 20 mL of distilled water and homogenized, and its pH was measured using a glass electrode [27]. The remaining feces was used for DNA analysis and kept at −80°C. The DNA was extracted using the QIAamp DNA stool kit (QIAGEN, Inc., Valencia, CA, USA) according to the manufacturer's instructions. The genomic DNA concentration was adjusted to 10 ng *μ*L^−1^ and stored at −25°C until analysis.

Real-time PCR analysis was performed as literature instruction, and the PCR primers that were used in this study are shown in Table 1. Real-time PCR amplification and detection were performed using an ABI prism 7000 sequence detection system (Applied Biosystems, Foster City, CA, USA). PCR was carried out in a reaction mixture with a final volume of 25 *μ*L containing 10 ng of template DNA, 12.5 *μ*L of SYBR Green master mix (Applied Biosystem, Foster City, CA, USA), and 4.5 *μ*L each of forward and reverse primers (0.5 *μ*L of forward and reverse primers for methanogen). Amplification consisted of 1 cycle of polymerase activation at 95°C for 10 min followed by 40 cycles of denaturing at 95°C for 15 s and 60°C for 30 s. The product size was confirmed by agarose gel electrophoresis after each determination. Standard DNA was prepared from the 16S rDNA fragment of *Escherichia coli* cloned into pCR 2.1 vector. Sample-derived standards for methanogen were prepared from the treatment pool set of community DNA [17]. The gene fragment encoding *mcrA* from cow rumen was cloned into pCR 2.1 vector. Standard DNA for fungi was prepared from a pure culture of ITS1 fragment of *Neocallimastix* sp. strain SR 6 cloned into pCR 2.1 vector as described in Lwin et al. [28]. PCR products from these cloned DNA were used as standards. Amplified standard DNAs were purified using a QIAquick PCR purification kit (QIAGEN, Inc., Valencia, CA, USA) and quantified with spectrophotometry (at 260 nm). The standards were serially diluted by 10-fold and were prepared just prior to real-time PCR. All measurements were performed in triplicate.

### 2.3. Amplification of Archaeal 16S rRNA and *mcrA* Genes

DNA samples from each individual animal were pooled into 1 portion for each animal breed. The *mcrA*-specific primers described in Luton et al. [31] and the archaeal 16S rRNA gene primers [30] (Table 1) were used for DNA amplification. The PCR reaction mixture (25 *μ*L) contained 1.0 *μ*L of template, 0.5 *μ*M of each primer, 200 *μ*M of a dNTP mixture, 1× Ex Taq buffer, 0.5 mg/mL BSA, and 0.625 units of Ex Taq polymerase. PCR was carried out on a thermal cycler (Dice TP 600; TaKaRa, Otsu, Japan) with the following conditions: initial denaturation at 95°C for 3 min for the archaeal 16S rRNA gene and 94°C for 2 min for the *mcrA* gene, denaturation at 94°C for 30 s, elongation at 72°C for 90 s for the 16S rRNA gene and 30 s for the *mcrA* gene, and a final extension at 72°C for 10 min. The annealing temperatures and the number of cycles are shown in Table 1. Following electrophoresis on 1.0% agarose gels in Tris-acetate EDTA buffer, PCR products were visualized by ethidium bromide staining.

### 2.4. Cloning and Sequencing

The PCR products were then cloned into the pCR 2.1 vector using the TA Cloning Kit (Invitrogen, Carlsbad, CA, USA). Positive transformants were randomly picked, and the cloned DNA fragments were sequenced, as described by Matsui et al. [32]. A homology search of archaeal 16S rRNA gene sequences and deduced amino acid sequences of *mcrA* was performed with the Blast N and Blast X programs [33]. Chimeric artifacts of PCR were checked with the CHECK_CHIMERA online program of the Ribosomal Database Project (RDP-II) and omitted from analysis [34].

### 2.5. Phylogenetic Analysis

The archaeal 16S rRNA genes and deduced amino acid sequences of *mcrA* gene were aligned with Clustal X ver. 2.0 [35], and phylogenetic trees were constructed using the neighbor-joining method [36]. The stability of the branches was analyzed with 1000 bootstrap replications. Operational taxonomic units (OTUs), richness observations (Chao 1), and Shannon-Wiener index (*H*′) were calculated using the DOTUR program [37]. A 98% sequence similarity criterion was employed for OTU of 16S rRNA gene sequence. A criterion for OTU of *mcrA* was calculated from correlation between *mcrA* sequence distance and 16S rRNA gene sequences distance obtained from following methanogens of 23 species from 7 orders including Methanoplasmatales proposed by Paul et al. [25]; accession numbers of *mcrA* gene and 16S rDNA are shown in the parenthesis after species name; order Methanobacteriales: *Methanobrevibacter gottschalkii *(EU919431/U55239), *Methanobrevibacter millerae* (EU919430/NR_042785), *Methanobrevibacter ruminantium *(AF414046/NR_042784), *Methanobrevibacter smihii* (NC_009515/AY196669), and *Methanobrevibacter woosei *(EU919432/NR_044788), order Methanomicrobiales: *Methanocorpusculum bavaricum* (AF414049/NR_042787),* Methanocorpusculum labreanum *(AAP20896/NR_074173), *Methanocorpusculum parvum* (AY260445/AY260435), *Methanoculleus bourgensis *(NC_018227/AY196674), *Methanofollis liminatans* (AF414041/Y16429), *Methanogenium thermophilum* (AB300783/M59129), *Methanomicrobium mobile *(AF414044/M59142), *Methanospirillum hungatei* (YP_503573/M60880), order Methanosarcinales: *Methanosarcina barkeri* (Y00158/NR_074253), *Methanosaeta concilii* (YP_004383383/NR_102903), order *Methanococcales*: *Methanocaldococcus jannaschii* (L77117/NR_074233), *Methanococcus vannielii* (P07961/NR_074175), and *Methanospaera stadtmanae *(YP_447374/JQ346752), order *Methanocellales*: *Methanocella arvoryzae *(AM114193/NR_074232), *Methanocella conradii *(YP_005380187/JN048683), order *Methanopyrales*: *Methanopyrus kandleri *(NP_613940/NR_074539), order Methanoplasmatales: Methanogenic archaeon CRM1 (GQ339872/GQ339875) and Methanogenic archaeon DCM1 (GQ339873/GQ339876). The correlation of the *mcrA* distance data to the 16S rRNA distance data gave an equation *Y* = 2.1944*X* (*R* ^2^ = 0.6196) when the line was forced through the origin. When 0.02 (criterion for 16S rRNA OTU) was plugged into *X*, *Y* = 0.0439. Therefore, the criterion of OTU of amino acid sequence of *mcrA* was determined as 95% similarity. Distances of protein and DNA were calculated with protodist and dnadist of Phylip package (ver. 3.68), respectively. The coverage was calculated from the following formula: coverage (%) = [1 − (*n*/*N*)] × 100, where *N* is the total number of clones and *n* is an OTU that consists of only 1 clone. The evenness (*E*) was calculated from the *H*′ using the following formula: *E* = *H*′/*H*_max⁡_ [38], where *H*_max⁡_ = 1*n*(*S*). LIBSHUFF analysis was used to calculate the statistical significance of the differences between the 2 libraries using the mothur program [39].

### 2.6. Statistical Analysis

The statistical analysis of pH and microbial population in the feces was performed using the Student's *t*-test. The significance was set at *P* < 0.05.

### 2.7. Nucleotide Sequence Accession Numbers

All nucleic acid sequences obtained in this study were deposited in the DNA Data Bank of Japan (DDBJ), European Molecular Biology Laboratory (EMBL), and GenBank databases, under accession numbers AB739303–AB739402 for 16S rRNA gene sequences and AB739403–AB739502 for *mcrA* gene sequences.

## 3. Results

### 3.1. Fecal pH and Microbial Population Densities

pH and microbial population density are shown in Table 2. The average pH of the fecal samples from horse was higher than that from pony (*P* > 0.05). The total bacterial population density was similar in horse and pony. Densities of anaerobic rumen fungi and methanogen populations were higher in horse than in pony (*P* > 0.05).

### 3.2. Phylogenetic Analysis of the *mcrA* Gene and the Archaeal 16S rRNA Gene

**Figure 1:**
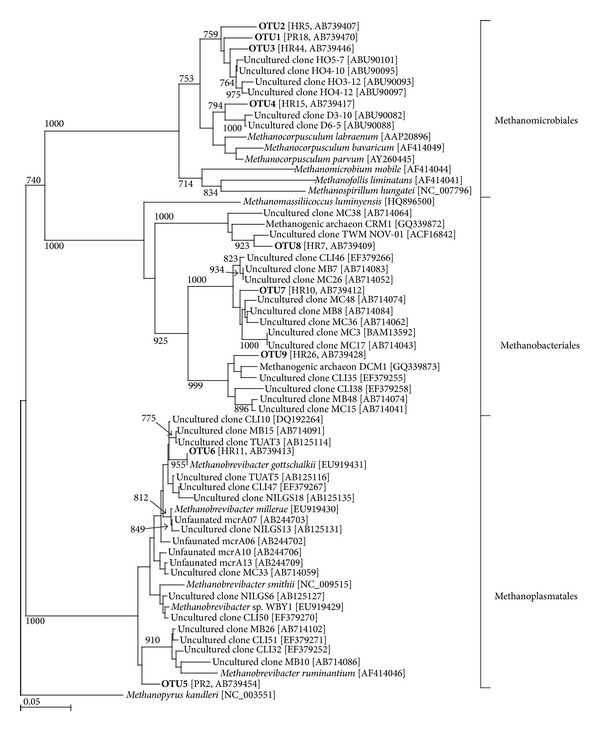
A phylogenetic tree showing the relationship between *mcrA* deduced amino acid sequences in the horse and pony. The tree was constructed using neighbor-joining analysis. The scale bar represents a 5% sequence divergence of amino acid sequence. Reference sequences were retrieved from the GenBank database, and their accession numbers are in brackets. OTU names from this study are labeled in bold. Representative clone name and its accession number are shown in brackets after OTU name. The *Methanopyrus kandleri* sequence was used as an outgroup to root the tree.

No chimeric sequence was found in the present study. A total of 50 clones were analyzed from the *mcrA* gene clone libraries of both horse and pony. The deduced amino acid sequences of the *mcrA* gene clones from horse and pony libraries were classified into 9 OTUs (Figure 1 and Table 3). The phylogenetic analysis showed that the OTUs were classified into 3 clades—Methanobacteriales, Methanomicrobiales, and Methanoplasmatales in both horse and pony (Figure 1). Most of the clones (4 OTUs and 46 clones, 92% in horse; 3 OTUs and 47 clones, 94% in pony) affiliated with Methanomicrobiales. Only 1 OTU was affiliated to genus *Methanobrevibacter* in both horse (OTU6) and pony (OTU5). OTU6 from the horse library showed 100% similarity to *Methanobrevibacter gottschalkii*. OTU5 showed 95% similarity to uncultured methanogens detected in the foregut of the tammar wallaby [40] (data not shown). The remaining 3 OTUs (OTU7, 8, and 9) in horse (6% of clones) and 1 OTU (OTU7) in pony (2% of clones) were classified as Methanoplasmatales (Table 3 and Figure 1). OTU9 showed a high similarity (96%) to the *mcrA* sequence of unidentified gut methanogenic archaeon DCM1 (published only in the database). Most of OTUs showed low similarity (less than 95%) to the identified methanogens (Table 3). OTU1, 2, 4, and 7 were commonly detected in both horse and pony library.

**Figure 2:**
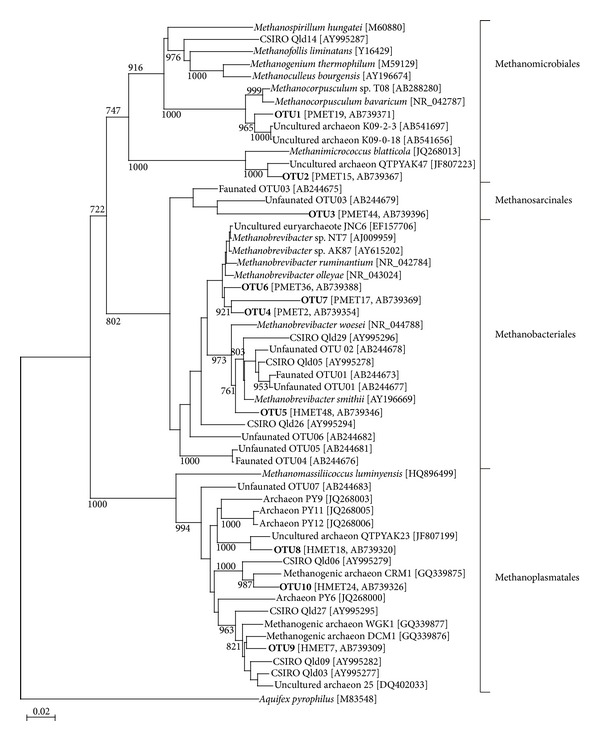
A phylogenetic tree showing the relationship between archaeal 16S rRNA sequences in the horse and pony. The tree was constructed using neighbor-joining analysis. The scale bar represents a 2% sequence divergence of DNA sequence. Reference sequences were retrieved from the GenBank database, and their accession numbers are in brackets. OTU names from this study are labeled in bold. Representative clone name and its accession number are shown in brackets after OTU name. The *Aquifex pyrophilus* sequence was used as an outgroup to root the tree.

Fifty clones of the archaeal 16S rRNA gene from the horse and pony libraries were analyzed. The cloned sequences from horse and pony libraries were classified into 10 OTUs (Figure 2 and Table 4). Phylogenetic analysis showed that the OTUs were classified into 4 clades (Figure 2). Similar to *mcrA* clone libraries, the majority of clones (32 clones, 64% in horse; 37 clones, 74% in pony) were affiliated with Methanomicrobiales (Table 4 and Figure 2). OTU2 did belong to Methanosarcinalesthat was not observed in *mcrA* clone library. OTU4, 5, and 6 showed a high similarity (97%–99%) to *Methanobrevibacter ruminantium*. These OTUs consisted of 18% and 14% of the total number of clones from the horse and the pony library, respectively. OTU8, 9 and 10 were classified into Methanoplasmatales clade (Figure 2). The clones from the horse library in these OTUs consisted of 10% of the total clone number, and 1 OTU from the pony library consisted of 6% of the total clone number (Table 3). OTU9 and OTU10 showed a high similarity (97% and 96%) to unidentified gut methanogenic archaeon DCM1 (GQ339876) and CRM1 (GQ339875) (published only in the database), respectively. OTU1, 2, 4, 6, and 8 were commonly detected in both horse and pony library.

In the analysis of the *mcrA* gene, the Shannon-Wiener index (*H*′), evenness, and Chao-1 species richness were higher in the horse library than in the pony library; however, LIBSHUFF analysis revealed that there was no significant difference in the diversity of *mcrA* genes in horse and pony (Table 5). Similar trends in *H*′ and evenness were observed in the analysis of the 16S rRNA gene. However, the LIBSHUFF analysis of the 16S rRNA gene library showed that there was a significant difference between the 2 libraries (*P* < 0.05). Lower coverages and higher Chao-1 species richness observed for the 16S rRNA gene clone libraries were higher than those observed for the *mcrA *gene clone libraries.

## 4. Discussion

A greater understanding of the microbial diversity of the hindgut is essential for improving the digestive process. The diversity of methanogens in the gastrointestinal tract of equine is also important for understanding the mitigation of methane emission. In the hindgut of nonruminants, methanogens use H_2_ and CO_2_ to produce methane [41]; however, methane production by monogastric animals is lower than methane production by ruminants. Additionally, among the monogastric animals, large herbivorous animals such as horses, mules, and asses produce a large amount of methane [42]. There is little information about the microbial ecosystem and methanogen diversity in equines. This study was conducted to establish further information about the methanogen diversity in horse and pony.

Morvan et al. [43] showed that the methanogen population was 10^4^ to 10^6^ cells per gram wet weight of cecal contents in horse. This study showed a similar density as that described by Morvan et al. [43]; however, there was no significant difference between horse and pony (Table 2).

Methanogens of the Methanomicrobiales are the most prevalent in the rumen of sheep (approximately 54%) and cattle (21–54%) [15, 16], and they were also dominant in Korean native cattle [12] and Murrah buffalo [44]. This study of a thoroughbred horse and Japanese native pony showed a similar tendency as that observed with the ruminal methanogen composition of sheep, cattle, and buffalo in previous studies.

Criterion for OTU assignment in the *mcrA* clone library was determined at 95% from correlation between *mcrA* distance and 16S rRNA gene distance. The criterion at 95% of *mcrA* corresponds to 98% of 16S rRNA gene. The analysis of *mcrA* revealed that most of the clones showed similarity less than 95% to the identified methanogen (Table 3). Furthermore, most of the clones from 16S rRNA gene library showed similarity less than 98% to the identified methanogen (Table 4). Therefore, these results suggest that most of the clones were derived from unidentified and novel species of methanogens.

The Methanoplasmatales were the second most dominant clade for both the *mcrA* and 16S rRNA gene analysis from the thoroughbred horse. The Methanoplasmatales clade represents a novel group of Archaea in the rumen of cattle [15], uncultured methanogens in the rumen of buffalo [44], uncultured Archaea in the rumen of cattle [13], or putative “new taxa” in the rumen of cattle [14]. Methanoplasmatales clade in horse also belongs to those new groups, and the OTUs in Methanoplasmatales clade showed 80%–92% similarity to amino acid sequence of *mcrA* genes and 91–97% similarity to 16S rRNA genes against identified or candidatus methanogen species in this study (Tables 3 and 4). Although OTU3 of 16S rRNA library showed 90% similarity to *Methanocorpusculum sinense *(FR749947), which is a member of Methanomicrobiales(Table 4), phylogenetic placement of the OTU was within the Methanobacteriales clade (Figure 2).

About half of the OTUs were commonly found in both breeds in the present study (Tables 3 and 4). Remaining OTUs were specifically found in each breed.

King et al. [13] showed that significant differences in 16S rRNA gene methanogen diversity were observed in different breeds of cows (Jersey versus Holstein) that were kept under the same dietary regimen. Similar to this report, a significant difference was found between horse and pony in the analysis of the 16S rRNA gene clone libraries by LIBSHUFF analysis (*P* < 0.05) (Table 5). Furthermore, the hindgut of horse was more diverse than that of pony. Phylogenetic analyses using 2 different gene clone libraries resulted in similar tendencies. However, the details of these distributions were different. The *mcrA* gene can be used for detecting more Methanomicrobialesspecies, while the 16S rRNA gene can be used to detect more *Methanobrevibacter* species. OTU that belongs to Methanosarcinales was detected only in the 16S rRNA clone library but not in the *mcrA* clone library. Thus using two different marker genes provides better resolution for the analysis of methanogen diversity.

## 5. Conclusions

The present study is the first report on the molecular diversities of methanogens in the hindgut of horse and pony based on *mcrA* and 16S rRNA gene clone library analysis. Although both animals harbored diverse group of methanogens, the composition was different (*P* < 0.05). The phylum Methanomicrobiales were the most abundant group in their hindgut. The clones affiliated to the phylum Methanoplasmatales which is recently proposed new phylum were also detected in the both libraries. Most of the clones obtained in this study were originated from unidentified methanogens, showing that the ecosystem is still unexplored environment. Isolation and characterization of the unidentified methanogens from hindgut of horse and pony should be done to clarify their function in the hindgut.

## Figures and Tables

**Table 1:** PCR primers used in real-time PCR assays and in clone library analysis.

Application	Target	Name (direction)	Sequence (5′→3′)	Annealing temperature (°C)	Number of cycles	Product size (bp)	Reference
Quantitative real-time PCR	Anaerobic fungi	q-pcr (f)	GAGGAAGTAAAAGTCGTAACAAGGTTTC	60	40	120	[29]
		q-pcr (r)	CAAATTCACAAAGGGTAGGATGATT				
	Methanogen	q-mcra (f)	TTCGGTGGATCDCARAGRGC	60	40	141	[23]
		q-mcra (r)	GBARGTCGWAWCCGTAGAATCC				
	Total bacteria	1114 (f)	CGGCAACGAGCGCAACCC	60	40	130	[29]
		1275 (r)	CCATTGTAGCAGGTG				

16S rRNA library	Methanogen	Met 86 (f)	GCTCAGTAACACGTGG	53	30	1254	[30]
		Met 1340 (r)	CGGTGTGTGCAAGGAG				

*mcrA* library	Methanogen	*mcrA*(f)	GGTGGTGTMGGATTCACACARTAYGCWACAGC	58	30	480	[31]
		*mcrA* (r)	TTCATTGCRTAGTTWGGRTAGTT				

f: forward; r: reverse.

**Table 2:** pH and population densities (copy numbers per gram wet weight of feces) of anaerobic rumen fungi, methanogen, and bacteria in feces of the horse and pony.

Item	Horse	Pony
pH	7.19 ± 0.38	6.79 ± 0.04
Total bacteria (×10^10^ copy/gram)	2.72 ± 0.81	2.31 ± 0.99
Methanogens (×10^6^ copy/gram)	11.30 ± 17.88	4.38 ± 1.48
Anaerobic rumen fungi (×10^5^ copy/gram)	2.31 ± 2.23	1.52 ± 0.80

**Table 3:** The number of clones and similarity of the deduced amino acid sequences of the *mcrA* gene of each operational taxonomic unit (OTU) to cultured methanogens in horse and pony.

OTUs	Nearest known methanogen*	Number of clones		
		Horse	Pony	Total
Methanomicrobiales				
OTU1	*Methanocorpusculum labreanum* [NC_008942] (93)	29	33	62
OTU2	*Methanocorpusculum labreanum* [NC_008942] (90)	10	13	23
OTU3	*Methanocorpusculum labreanum* [NC_008942] (93)	6	0	6
OTU4	*Methanocorpusculum labreanum* [NC_008942] (94)	1	1	2

Methanobacteriales				
OTU5	*Methanobrevibacter smithii* [DQ251046] (94)	0	2	2
OTU6	*Methanobrevibacter gottschalkii* [ACK56066] (100)	1	0	1

Methanoplasmatales				
OTU7	Candidatus *Methanomethylophilus alvus* [KC412011] (80)	1	1	2
OTU8	Candidatus *Methanomethylophilus alvus* [KC412011] (92)	1	0	1
OTU9	Candidatus *Methanomethylophilus alvus* [KC412011] (83)	1	0	1

*Number in brackets and in parenthesis is accession number and similarity value (%), respectively.

**Table 4:** The number of clones and similarity of the archaeal 16S rRNA gene sequences of each operational taxonomic unit (OTU) to cultured methanogens in horse and pony.

OTUs	Nearest known methanogen*	Number of clones		
		Horse	Pony	Total
Methanomicrobiales				
OTU1	*Methanocorpusculum labreanum* [NR_074173] (96)	32	37	69

Methanosarcinales				
OTU2	*Methanomicrococcus blatticola* [AJ238002] (94)	4	1	5

Methanobacteriales				
OTU3	*Methanocorpusculum sinense* [FR749947] (90)	0	1	1
OTU4	*Methanobrevibacter ruminantium* [CP001719] (97)	1	5	6
OTU5	*Methanobrevibacter smithii *[NR_074235] (97)	6	0	6
OTU6	*Methanobrevibacter ruminantium* [CP001719] (98)	2	2	4
OTU7	*Methanobrevibacter ruminantium* [CP001719] (94)	0	1	1

Methanoplasmatales				
OTU8	Candidatus *Methanomethylophilus alvus* [KC412010] (91)	1	3	4
OTU9	Candidatus *Methanomethylophilus alvus* [KC412010] (93)	2	0	2
OTU10	Candidatus *Methanomethylophilus alvus* [KC412010] (97)	2	0	2

*Number in brackets and in parenthesis is accession number and similarity value (%), respectively.

**Table 5:** General information and diversity indices of the *mcrA* gene and archaeal 16S rRNA gene clone libraries recovered from microbial populations in the fecal contents of horse and pony.

Item	*mcrA* gene		16S rRNA gene	
	Horse	Pony	Horse	Pony
Number of clones	50	50	50	50
Number of OTUs	8	5	8	7
Coverage (%)	37.5	86	75	57
Shannon-Wiener index (*H*′)	1.28	0.91	1.29	0.99
Evenness	0.33	0.23	0.33	0.25
Chao-1 species richness	18	7	12	13
LIBSHUFF analysis	Ns		*P* < 0.05	

Ns: not significant.
